# Does the Strategy of Risk Group Testing for Hepatitis C Hit the Target?

**DOI:** 10.3389/fphar.2017.00437

**Published:** 2017-06-30

**Authors:** Mirjana R. Jovanovic, Aleksandar Miljatovic, Laslo Puskas, Slobodan Kapor, Dijana L. Puskas

**Affiliations:** ^1^Psychiatric Clinic, Clinical Center KragujevacKragujevac, Serbia; ^2^Department for Psychiatry, Faculty of Medical Sciences, University of KragujevacKragujevac, Serbia; ^3^Psychiatric Clinic, Clinical Center ZvezdaraBelgrade, Serbia; ^4^Faculty of Medicine, University of BelgradeBelgrade, Serbia; ^5^Faculty of Special Rehabilitation and Education, University of BelgradeBelgrade, Serbia

**Keywords:** hepatitis C infection, testing strategies, risk groups, cost-effectiveness

## Abstract

In the European Union, it is estimated that there are 5.5 million individuals with chronic infection of hepatitis C. Intravenous drug abuse is undoubtedly the key source of the hepatitis C epidemic in Europe and the most efficient mode of transmission of HCV infections (primarily due to short incubation time, but also because the virus is introduced directly into the blood stream with the infected needle). Potentially high-risk and vulnerable populations in Europe (and the world) include immigrants, prisoners, sex workers, men having sex with men, individuals infected with HIV, psychoactive substance users etc. Since there is a lack of direct evidence of clinical benefits of HCV testing, decisions related to testing are made based on indirect evidence. Clinical practice has shown that HCV antibody tests are mostly adequate for identification of HCV infection, but the problem is that this testing strategy does not hit the target. As a result of this health care system strategy, a large number of infected patients remain undetected or they are diagnosed late. There is only a vague link between screening and treatment outcomes since there is a lack of evidence on transmission risks, multiple causes, risk behavior, ways of reaching screening decisions, treatment efficiency, etc. According to results of limited number of studies it can be concluded that there is a need to develop targeted programmes for detection of HCV and other infections, but there also a need to decrease potential harms.

## Some facts about HCV presence

In 2014, 35,321 new cases of hepatitis C were reported from 28 EU/EEA member states, while a “crude” rate was 8.8 cases per 100,000 population (European Centre for Disease Prevention and Control, 2013). Out of these cases, 1.3% were classified as acute, 13.3% as chronic, 74.7% as “unknown,” and 10.7% were not classified. Intravenous drug abuse is undoubtedly the key source of the hepatitis C epidemic in Europe and the most efficient mode of transmission of HCV infections (primarily due to short incubation time, but also because the virus is introduced directly into the blood stream with the infected needle).

The prevalence of HCV among drug addicts is between 60 and 80% which is in direct correlation to the period of psychoactive substance abuse. This way HCV infection is transmitted 10 times faster and more efficiently than HIV infection (Mosley et al., 2005; Wang et al., 2016b).

In the European Union, it is estimated that there are 5.5 million individuals with chronic infection. Intravenous drug use is the key issue in dispersion of HCV infection in Europe—national estimates of antibody-prevalence range from 15 to 84% (European Monitoring Centre for Drugs Drug Addiction, 2016).

Potentially high-risk and vulnerable populations in Europe (and the world) include immigrants, prisoners, sex workers, men having sex with men, individuals infected with HIV, psychoactive substance users etc. (Forouzanfar et al., 2016).

In Serbia, which geographically belongs to the Western Balkans, the situation is similar to other countries in the region—epidemiological characteristics of HCV infection have not been studied reliably since there is no continuous and comprehensive disease monitoring. Moreover, there are only few limited studies on socio-economic background of this disease in Serbia. Regardless the advancement in the disease treatment, it is of vital importance to have epidemiological and pharmacological data in order to make the plan of prevention and control more efficient (Mitrovic et al., 2015).

Based on limited range studies, the prevalence of HCV in Serbia is higher than 1% (i.e., the estimated prevalence in general population is 1.13% (95% CI: 1.0–1.26%) (European Centre for Disease Prevention and Control, 2013), while in Europe it is about 1.5% (Cooke et al., 2013). In our population, the most common HCV genotypes are genotype 1 (63%) and genotype 3 (27%), while genotype 2 and 4 account for 7 and 3% of the cases, respectively. Genotypes 5 and 6 have not been registered (Mitrovic et al., 2015).

Jakovljević et al. carried out a study in 2013 which compared the costs of patients with genotypes 1 or 4 (group I) and patients with genotypes 2 or 3 (group II). It showed that the patients with genotypes 1 and 4 caused significantly higher direct medical costs which did not include medicine purchase costs. When the costs of the consumed pegylated interferon alfa plus ribavirin were added, the expenses moved toward patients with genotype 2 or 3 infection. Finally, when indirect costs (e.g., lost productivity costs) were taken into account, the total costs were even 25% higher among patients with genotype group 2. The conclusion was that an average patient belonging to either of the groups incurred €18,121.04 costs per protocol for the treatment period less than a year (Jakovljevic et al., 2013).

To make a comparison, the estimates from the Health Protection Agency (HPA) in Great Britain (Hepatitis, 2013) show, based on the research carried out by the National Institute for Health and Clinical Excellence (NICE), that the cost of antiviral treatment of individuals with hepatitis C varies between £6,246 for those requiring 24-week treatment (mainly genotypes 2 and 3) and £12,741(14,714.80 euros) for those requiring a standard treatment of 48 weeks (mainly genotype 1) (Ramsay, 2011).

This means that the treatment of an individual infected with hepatitis C in Great Britain is almost €3,500 cheaper than in Serbia. From 2006 to 2014, GDP in Serbia ranged from $3,700 to 4,300, while in Great Britain it was $39,511^1^.

The costs given by the HPA are in compliance with the costs reported in 2011 by the British National Formulary for peginterferon alfa-2a (Pegasys), peginterferon alfa-2b (ViraferonPeg), and ribavirin (Copegus, Rebetol) (British National Formulary, 2015-2016), including the treatment monitoring costs taken over from Hartwell et al. (2011).

However, the budget impact of HCV treatment has been significant. Classic treatments resulted in more significant side effects and they were less effective than newer treatments. The most important issue (always) is the price. Health stakeholders should use scientific information to increase the efficiency and availability of treatment and reduce costs. Some studies show that the prevalence growths associated with the increase in annual health cost are, for example, from £82.7 m in 2012 to £115 m in 2035. Also, productivity losses were estimated to rise from between £184 and £367 m in 2010 to between £210 and £427 m, in 2035 (Hepatitis, 2013).

## HCV screening and outcomes

Hepatitis C is an important public health issue not only in Europe but all around the world, considering high costs associated with morbidity and mortality (ECDC, 2015; Wang et al., 2016a). The Global Burden of Disease (GBD) is 42% for mortality as a result of liver cancer caused by hepatitis C. Approximately 700,000 people die each year from the consequences of this infection (Lozano et al., 2012).

Monitoring does not always give a clear picture of the situation in a particular country. For example, it is estimated that 2.2–3.2 million people are chronically infected with hepatitis C in the USA, but half of them is unaware of that (CDC, 1998).

Despite the limitations of routine monitoring of HCV infection, the available data clearly indicate that the largest number of reported cases was associated with drug injecting and other groups at risk.

**Figure 1:**
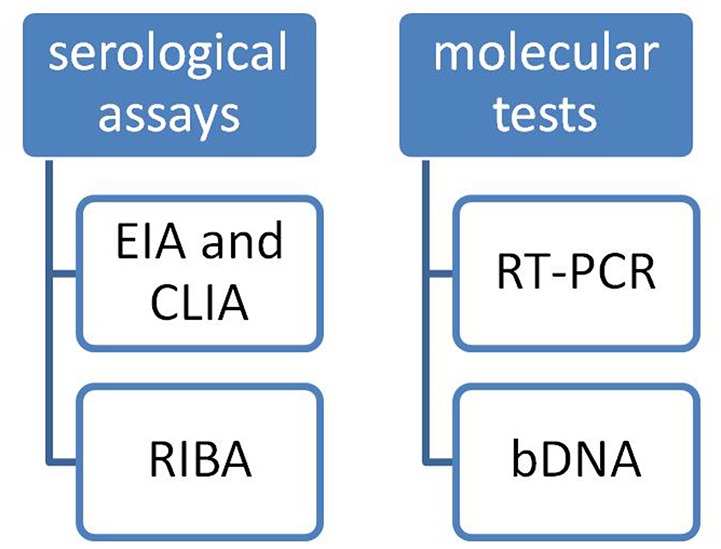
HCV tests.

There are two kinds of **tests** typically used to **diagnose** HCV infection (Figure 1):

1. **Serological assays** that detect antibody to hepatitis C virus (anti-HCV);
2. **Molecular assays** that detect, quantify, and/or characterize HCV RNA genomes

Serological assays can be subdivided into:

1. Screening tests for anti-HCV such as EIA and CLIA (Chemiluminescence Immuno Assay).
2. And supplemental tests such as RIBA (Recombinant Immunoblot Assay) test.

In the 1980s first generations of **serological assays** were developed. The first-generation anti-HCV EIA detected antibodies 12–26 weeks after exposure to infection, thus creating a long window period of infectivity. With the second generation of tests the window period of infectivity was reduced to 10–24 weeks. So far three generations of serological assays have been developed in order to improve sensitivity and specificity (Marwaha and Sachdev, 2014). Nowadays, the third-generation of EIA assays is used to detect antibodies against reconfigured hepatitis C proteins: C, NS3, and NS4, as well as NS5A antigen which the previous generation of assays did not contain (Gretch, 1997).

The third-generation EIA brought about a new reduction in the window period by a week. Despite many attempts to increase sensitivity of assays, the problem of serology negative but “infectious” window period remained, which with the second-generation assays was 82 days (Busch et al., 1995) and with the third-generation remained around 66 days (Couroucé and Pillonel, 1996).

Combination antigen-antibody assays were introduced few years ago, when, two markers of the same infection were detected at the same time. These assays are called “fourth-generation” or “antigen-antibody combo” tests. They are suitable for testing in blood banks where a large numbers of samples need to be tested in a short period of time. The average window period for these assays is 26.8 days (CDC, 2013).

RIBA tests are used to confirm a positive enzyme immunoassay, while the same serum samples can be used. RIBA use recombinant antigens and synthetic peptides similar to EIA. They are in immunoblot format, so that they can detect antibodies for specific proteins.

The test result is reported positive if antibodies to two or more antigens are detected, inconclusive or indeterminate if antibodies to one antigen are detected, or negative. These tests are recommended to be used primarily for patients at low risk for HCV like volunteer blood donors (Alter et al., 2003; Narciso-Schiavon et al., 2008). A positive anti-HCV antibody test does not distinguish between a current and a past infection, but it indicates the need for further medical evaluation (Fonseca et al., 2011). Among immunocompromised individuals, serological tests can have false-negative results, for example with HIV-infected patients, with patients with renal insufficiency and with patients with essential mixed cryoglobulinemia caused by HCV (Alter et al., 2003) (Table 1).

**Table 1:** Testing for HCV infection.

**Test result**	**Interpretation**	**Further action**
HCV antibody non-reactive	No HCV antibody detected	Sample may be non-reactive for HCV antibody. No further action is required. If there was a recent exposure, test for HCV RNA.
HCV antibody reactive	Presumptive HCV infection	A repeatedly reactive result indicates current HCV infection, past HCV infection that has resolved or biologic false positivity for HCV antibody. Test for HCV RNA is order to identify current infection.
HCV antibody reactive	Current HCV infection	Organize testing with appropriate counseling and link the tested person to future treatment.
HCV RNA detected		
HCV antibody reactive	No current HCV infection	In most cases, no further action is needed.
HCV RNA not detected		If there is desired distinction between true positivity and biologic false positivity for HCV antibody, and if the sample is repeatedly reactive in the initial test, test with another HCV antibody assay.
		In certain situations, continue with HCV RNA testing and appropriate counseling.

*CDC. Testing for HCV infection: An update of guidance for clinicians and laboratorians. MMWR 2013; 62 (Gretch, 1997)*.

**Qualitative molecular tests** are based on RT-PCR technique. These tests have a detection limit of 50 IU/ml and they are used to confirm viremia and to monitor treatment response^2^. If the test result is positive, there is an active infection. Qualitative PCR tests are also used with EIA negative patients with suspected acute infection, with patients diagnosed with hepatitis of unknown cause, as well as with those with known causes of false-negative results of antibody tests.

Qualitative molecular tests, PCR, and bDNA (branched DNA assay) are used to monitor anti-HCV treatment (Alter et al., 2003).

A very significant non-specific alanine aminotransferase measurement (ALT) test used to monitor infections and treatment effectiveness should be also mentioned here (Alter et al., 2003).

The AASLD (American Association of the Study of Liver Diseases) and the IDSA (Infectious Disease Society of America) strongly recommend annual HCV testing for persons who inject drugs and for HIV-seropositive men who have unprotected sex with men^1^.

## What about testing strategies?

Since there is a lack of direct evidence of clinical benefits of HCV testing, decisions related to testing are made based on indirect evidence (Chou et al., 2012). Furthermore, clinical practice has shown that HCV antibody tests are adequate for identification of HCV infection. The problem is that this testing strategy does not hit the target. As a result of this health care system strategy, a large number of infected patients remains undetected or are diagnosed late. Potentially, these patients are permanent sources of infection, which is very important especially for groups at risk. This way, our understanding of the actual risks and the real dimensions of this problem remains incomplete. In order to make screening more effective, besides strategies to identify HCV infected individuals, there should be strategies for further actions including counseling, education, medical treatment, physiological, and psychiatric support etc., with the aim to improve treatment outcomes. At this time, there is only a vague link between screening and treatment outcomes since there is a lack of evidence on transmission risks, multiple causes, risk behavior, ways of reaching screening decisions, treatment efficiency, etc.

Retrospective studies that analyzed strategies that target several risk factors showed sensitivity of over 90% and the need to test up to 20 people in order to identify one HCV-infected person (Gunn et al., 2003; Zuure et al., 2010).

However, there have been no prospective studies to compare different screening strategies or consider a new (alternative) approach to screening or possible outcomes. In the USA, epidemiological data show that about two-thirds of people with chronic hepatitis C were born between 1945 and 1965. Birth-cohort screening may be a useful future screening strategy. The only published birth-cohort is a cost-effectiveness study from 2012 (Rein et al., 2012).

Some studies published in the last decade suggest the knowledge of being infected reduces risk behavior of some patients (Hagan et al., 2006; Scognamiglio et al., 2007; Trepka et al., 2007), but prospective studies show that this behavior is not sustained over time (Tsui et al., 2009).

Nevertheless, there are many uncertainties concerning potential harms and benefits of HCV testing. There is a need to study psychological aspects of testing such as fear, anxiety, acute stress reaction, impact on quality of life, impact on partner relationships, family, and social relations, etc. There is also a question whether a wider concept of counseling would contribute to reduction of potentially harmful influences of the given factors.

Testing efficiency and cost-effectiveness are present additional problems. How reasonable is to repeat rapid antibody detection tests?

Patient testing as part of PAS addiction treatment programmes is a good way to target chronically infected individuals which enables implementation of potentially new approaches to treatment which might become more efficient that the existing ones (Afdhal et al., 2013; Frimpong, 2013).

A research carried out within the programme for community-based treatment of addicts by the National Drug Abuse Treatment Clinical Trials Network (CTN), showed that only 28% of the USA programmes offered HCV testing as part of their programme or in the nearest reference center (Pollack and D'Aunno, 2010; Bini et al., 2012). The latest researches show a significant reduction in HIV and HCV testing within opiate addiction treatment programmes between 2005 and 2011 and a significant increase of testing within public treatment programmes (D'Aunno et al., 2014), which suggests that scarce resources can play an important role in deciding whether to invest into private-profitable or unprofitable programmes.

Between 2003 and 2014, Serbia received ~$30 million from the Global Fund for development and implementation of HIV and HCV prevention and treatment in Serbia. However, as a middle income country, Serbia lost the funding abruptly when its HIV burden was estimated as “moderate” (The Global Fund, 2015). Furthermore, in 2012 Serbia was removed from the list of countries eligible for support in 2013 (Jakovljevic et al., 2017).

In 26 opiate addiction treatment centers in Serbia, HCV testing was drastically reduced because they were no longer financed by the Global Fund. The testing programmes included rapid tests within the treatment centers or at the nearest reference centers. Since 2014, the number of tests has been constantly decreasing (Jakovljević and Jovanović, 2011).

The World Health Organization states that the Global Fund supports comprehensive harm reduction packages that include preventive activities, testing and treatment of hepatitis C (World Health Organization, 2014).

Based on the available evidence, on-site rapid HIV and hepatitis C testing at addiction treatment centers is an excellent investment in public health (Jakovljevic et al., 2016).

Legal authorities should identify the ways to improve and implement on-site HCV and HIV rapid testing at addiction treatment centers and ensure that the individuals with positive results proceed to further treatment and further evaluation (Schackman et al., 2015).

One of major concerns is liver biopsy which is an invasive procedure with potentially serious adverse effects which is still the only reliable method to determine the histological state of the liver in HCV-infected individuals. A possible alternative would be to develop non-invasive techniques and tests to determine the stage of the disease (Chou et al., 2012). However, further comparative studies are needed to determine the significance of the liver biopsy in relation to further treatment courses.

There is also the issue of testing and education of specific groups and the impact of testing on the public health.

Testing and education should not be limited only to groups at risk such as PAS users, it should include other groups in need for immunization such as vaccination of alcoholics against hepatitis A and B and implementation of a known HCV detection strategy.

Hepatitis C virus infection is the most prevalent infection among intravenous drug users. According to the study conducted in Serbia in 2008, the prevalence among intravenous drug users was 69% in Belgrade, 50% in Niš, and 45% in Novi Sad.

The same study showed that after voluntary, free of charge and confidential testing on HCV, HIV and syphilis, more than 55.3% of the tested individuals in Belgrade and 43.5% in Niš failed to return for their test results. (Mickovski, 2010). The future studies should take into account the cost of testing, motivation and other psychological characteristics of the studied patients, as well as the outcomes of these interventions.

Studies conducted in other countries also show that only a small number of people injecting drugs returns for their results (Hagan et al., 2006; McDonald et al., 2010). Since this is a high-risk population, an adequate strategic approach is needed to enable frequent testing, preferably free of charge and an easy way to get to an infectologist and further treatment. To be more precise, testing should be done in places where the therapy is administered.

Although screening can positively identify adults with chronic HCV infection, more research is needed to understand the effects of different screening strategies on the clinical outcome. The evidence on effects of knowledge of HCV status, counseling, and vaccination of HCV diagnosed patients are still scarce. There should also be more studies on interventions that could successfully prevent vertical transmission. A comprehensive assessment of benefits and harms of screening includes evaluation of the effectiveness of antiviral regimes is also necessary.

Furthermore, there is a need for a cost-effectiveness study of the fourth-generation HCV antigen and antibody assay (combination EIA) two HCV in the same assay. Molecular testing for HCV-RNA using nucleic acid amplification technology (NAT) is today the most sensitive assay that shortens the window period to only 4 days. Implementation of NAT in many developed countries in the world has resulted in dramatic reductions in transfusion transmitted HCV infections, so now the relative risk is <1 per million donations (CDC, 2013).

If we bear in mind all the facts stated above, it becomes obvious that future studies will have to consider testing costs in relation to potential benefits in specific, high-risk populations.

## Conclusion

The current hepatitis C testing strategy is not efficient, as concluded by numerous studies.

Research, development, validation, and cost-effectiveness studies should yield best practices for detecting HCV viremia and for developing new possibilities to distinguish between people with resolved HCV infection and those with biologic false positivity for HCV antibody, in whom HCV RNA has not been detected. The results of these studies should provide comprehensive guidelines for testing, reporting, and clinical management and improve definitions for disease reporting and surveillance. (Gregory and Dodd, 2009; Marwaha and Sachdev, 2014).

Based on the presented facts, it can be concluded that there is a need to develop targeted programmes for detection of HCV and other infections, but there also a need to decrease potential harms. Furthermore, public health programmes have to be made according to the local epidemiological picture and taking into account new research evidence on efficiency and effectiveness. Also, there is a need to include innovation in health system products, processes, and delivery systems and to optimize the performance of medical care through better understanding causes and courses of HCV infection and also, treatment consequences.

## Author contributions

All authors listed, have made substantial, direct and intellectual contribution to the work, and approved it for publication.

### Conflict of interest statement

The authors declare that the research was conducted in the absence of any commercial or financial relationships that could be construed as a potential conflict of interest. The reviewers MI, BB and the authors declared their shared affiliation, and the handling editor states that the process met the standards of a fair and objective review.

## Footnotes

^1^http://www.penzin.rs/koliko-drzave-sveta-prave-novca-po-stanovniku-a-koliko-srbija/

^2^Available online at: http://www.hcvguidelines.org/printpdf/12
